# BASIC SCREENING SURVEY IN DETROIT SENIOR CENTERS

**DOI:** 10.1093/geroni/igad104.2777

**Published:** 2023-12-21

**Authors:** April Koternamski, Divesh Byrappagari, Judith Jones

**Affiliations:** University of Detroit Mercy School of Dentistry, Detroit, Michigan, United States; University of Detroit Mercy School of Dentistry, Detroit, Michigan, United States; University of Detroit Mercy School of Dentistry, Detroit, Michigan, United States

## Abstract

The objective of the Senior Oral Health Equity Project (SOHEP) Basic Screening Survey (BSS) was to determine the oral health status of seniors 55yrs and older in Detroit senior centers. The ASTDD BSS tool was used to collect the data. The screenings were conducted in two local senior centers to determine the prevalence of dental disease as well as dental care needs. The data were collected using Qualtrics© and analyzed using SPSS©. Majority of the seniors (n=67) screened were African American (88.9%), female (79.1%) and between the ages of 65 and 85 yrs. (89.6%). Results showed that there is a high prevalence of tooth loss (6 or more teeth – 59.6%), untreated decay (38.8%), need for periodontal care (52.2%) and need for dental care (43.3%). The results suggest that the senior center participants have limited access to dental care. There is a need to develop preventive dental programs for seniors where they congregate and live.

